# Understanding Fc Receptor Involvement in Inflammatory Diseases: From Mechanisms to New Therapeutic Tools

**DOI:** 10.3389/fimmu.2019.00811

**Published:** 2019-04-12

**Authors:** Sanae Ben Mkaddem, Marc Benhamou, Renato C. Monteiro

**Affiliations:** ^1^INSERM U1149, Centre de Recherche sur l'Inflammation, Paris, France; ^2^CNRS ERL8252, Paris, France; ^3^Faculté de Médecine, Université Paris Diderot, Sorbonne Paris Cité, Site Xavier Bichat, Paris, France; ^4^Inflamex Laboratory of Excellence, Paris, France; ^5^Service d'Immunologie, DHU Fire, Hôpital Bichat-Claude Bernard, Assistance Publique de Paris, Paris, France

**Keywords:** immunoglobilins, Fc receptor, antibody treatment, signaling/signaling pathways, inflammatory diseases

## Abstract

Fc receptors (FcRs) belong to the ITAM-associated receptor family. FcRs control the humoral and innate immunity which are essential for appropriate responses to infections and prevention of chronic inflammation or auto-immune diseases. Following their crosslinking by immune complexes, FcRs play various roles such as modulation of the immune response by released cytokines or of phagocytosis. Here, we review FcR involvement in pathologies leading notably to altered intracellular signaling with functionally relevant consequences to the host, and targeting of Fc receptors as therapeutic approaches. Special emphasis will be given to some FcRs, such as the FcαRI, the FcγRIIA and the FcγRIIIA, which behave like the ancient god Janus depending on the ITAM motif to inhibit or activate immune responses depending on their targeting by monomeric/dimeric immunoglobulins or by immune complexes. This ITAM duality has been recently defined as inhibitory or activating ITAM (ITAMi or ITAMa) which are controlled by Src family kinases. Involvement of various ITAM-bearing FcRs observed during infectious or autoimmune diseases is associated with allelic variants, changes in ligand binding ability responsible for host defense perturbation. During auto-immune diseases such as rheumatoid arthritis, lupus or immune thrombocytopenia, the autoantibodies and immune complexes lead to inflammation through FcR aggregation. We will discuss the role of FcRs in autoimmune diseases, and focus on novel approaches to target FcRs for resolution of antibody-mediated autoimmunity. We will finally also discuss the down-regulation of FcR functionality as a therapeutic approach for autoimmune diseases.

## Fc receptor modes of action

Immunoglobulin Fc receptors (FcRs) are membrane molecules expressed by several hematopoietic cells that recognize the Fc region of several immunoglobulin (Ig) classes and subclasses. We distinguish FcR for IgG (FcγRI/CD64, FcγRII/CD32, and FcγRIII/CD16), IgE (FcϵRI), IgA (FcαRI/CD89), IgM (FcμR), and IgA/IgM (Fcα/μR). Several other receptors expressed on different cell types also bind Ig molecules: neonatal FcR for IgG (FcRn) on intestinal epithelium, placenta, and endothelium, low affinity FcϵR (FcϵRII/CD23) on B cells and macrophages, and polymeric Ig receptor (pIgR) on mucosal epithelium (1–3).

The function of antibodies depends on one hand on their ability to recognize antigenic epitopes and, on the other hand, on their dynamic flexibility and their capacity to interact with their cognate FcRs. Engagement of FcRs expressed by leukocytes initiates a number of pro-inflammatory, anti-inflammatory, and immune modulatory functions in the host adaptive immune responses leading to protection but sometimes also to disease.

**Figure 1:**
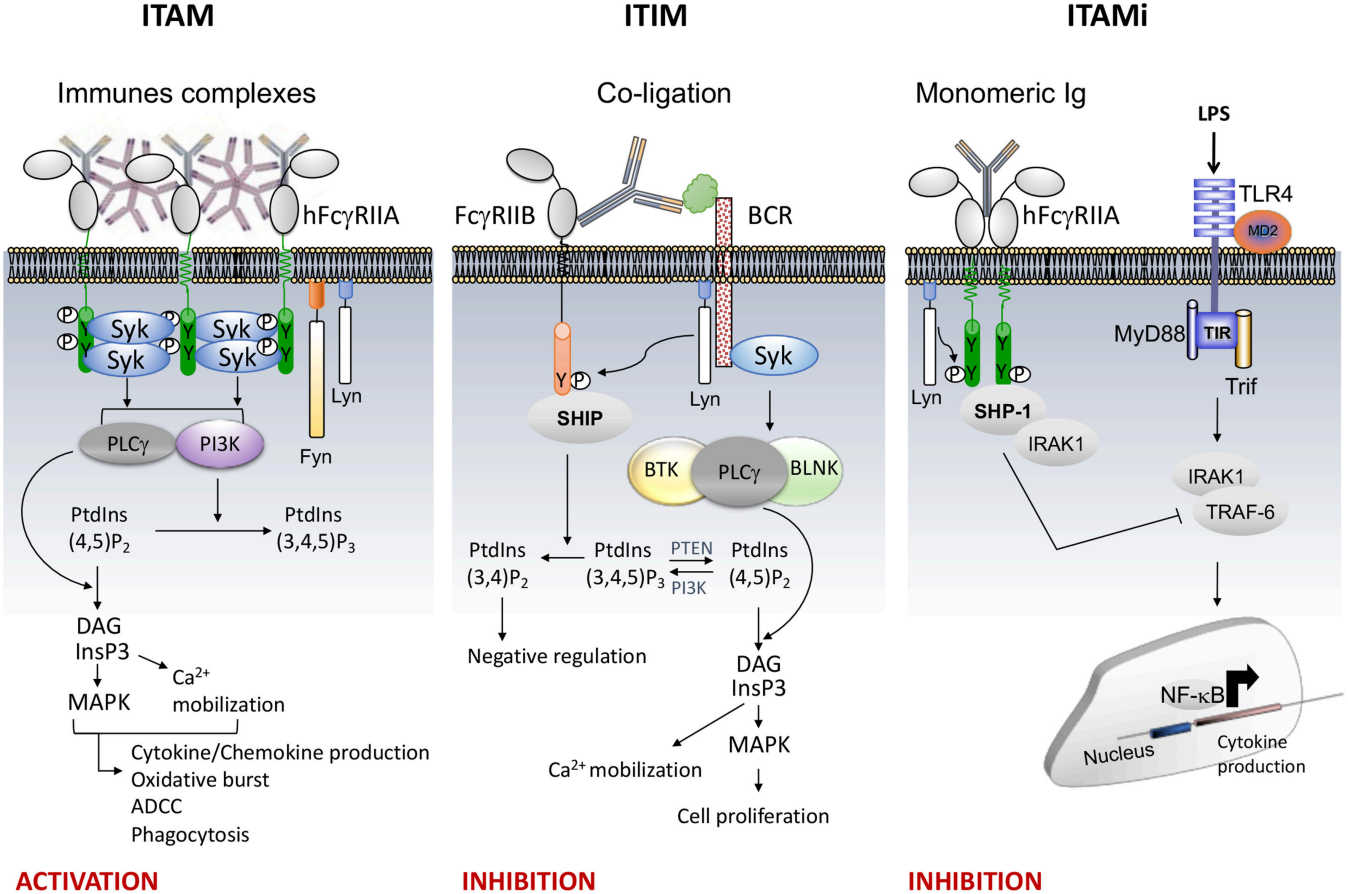
FcR signaling (e.g., FcγRII). **(Left)**, the aggregation by an immune complex of FcR bearing ITAM motif (e.g. FcγRIIA) induces phosphorylation of the two ITAM tyrosine residues by Src kinases Lyn and Fyn responsible for recruitment and phosphorylation of Syk inducing cellular activation through PLCγ and PI3K signaling pathways. The PLCγ converts PI(4,5)P_2_ into IP_3_ and DAG. IP_3_, a soluble inositol phosphate, leads to Ca^2+^ mobilization while DAG activate MAPK.PI3K converts PI(4,5)P_2_ to PI(3,4,5)P_3_ allowing recruitment of signal intermediates through their pleckstrin homology (PH) domain **(Middle)**, co-ligation between an activating heterologous receptor (e.g., the BCR) and the inhibitory FcR (i.e., FcγRIIB) induces phosphorylation of the tyrosine present within the ITIM motif by Lyn (5), leading to the phosphorylation and recruitment of phosphatases (SHIP or SHP). The phosphatases PTEN and SHIP1/2 regulate cellular levels of PI(3,4,5)P_3_ by hydrolyzing it to PI(4,5)P_2_ and PI(3,4)P_2_, respectively. These dephosphorylations inhibit cell proliferation. **(Right)**, monovalent targeting of FcR bearing ITAM motif (e.g., FcγRIIA) induces the phosphorylation of the last tyrosine residue of the ITAM motif by Lyn responsible for transient recruitment of Syk followed by that of SHP-1 which abrogates the activation signal.

Several FcRs require the Immunoreceptor Tyrosine-based Activation Motif (ITAM; with the sequence Yxx[L/I]x_(6−8)_Yxx[L/I]) present in the cytoplasmic tail of the receptor or of associated subunits (FcRγ or FcεRIβ chain) to induce cell signaling. ITAM-mediated functions include phagocytosis, degranulation, antibody-dependent cellular cytotoxicity (ADCC), cytokine, lipid mediator and superoxide production, all of which depend on the cell type and on outside-in signals induced by the ligand. Engagement of the type I FcRs by immune complexes, induces receptor aggregation followed by activation and recruitment of Src family kinases (SFKs), such as Lyn and Fyn (4). The former induces the phosphorylation of the conserved tyrosines in the ITAM motif, followed by activation and recruitment of the tyrosine kinase Syk. This process activates various proteins involved in cell response, such as Phospholipase C gamma 1 (PLCγ), Bruton's tyrosine kinase (Btk), guanine nucleotide exchange factor Vav and phosphoinositide 3-kinase (PI3K). Hydrolysis of phosphatidylinositol 4,5-bisphosphate (PtdIns(4,5) P_2_) by PLCγ generates inositol 1,4,5-trisphosphate (IP_3_) and diacylglycerol (DAG) leading to calcium mobilization and protein kinase C (PKC) activation, respectively. Calcium influx and PKC activation promote cell responses such as degranulation and cytokine production. Vav plays also an important role in actin cytoskeleton remodeling to control phagocytosis and superoxide production by NADPH oxidase. PI3K catalyzes the phosphorylation of PtdIns(4,5)P_2_ into PtdIns(3,4,5)P_3_ in the plasma membrane. Pleckstrin homology domains contained in proteins such as PLCγ, GRB2-associated-binding protein 2 (Gab2), protein kinase B (PKB/Akt) and Btk, bind PtdIns(3,4,5)P_3_ thus recruiting them at the inner leaflet of the plasma membrane promoting their phosphorylation and activation (Figure 1, left).

The activation of ITAM-bearing immune receptors can be retro-controlled by ITIM-bearing inhibitory FcRs such as the FcγRIIB. The ITIM motif is defined by a single [I/V/L/S]xYxx[L/V] sequence. However, inhibition of cell activation by this motif requires co-ligation between the inhibitory and heterologous activating receptors by immune complexes promoting the recruitment of inositol phosphatases (SHIP-1 and SHIP-2) (6) (Figure 1, Middle). Another inhibitory mechanism has been recently identified that involves ITAM itself. Indeed, following low avidity ligand interactions, ITAM-bearing FcRs induce a sustained inhibitory signal without co-ligation with heterologous receptors. This mechanism was involved in the maintenance of immune homeostasis (7–14). We named this ITAM-mediated inhibitory signal, ITAMi. It has been shown that several low affinity receptors, such as FcαRI, FcγRIIA and FcγRIIIA, can function as such bi-functional receptors to induce either activating or inhibitory signals, a property that can be exploited to reduce the susceptibility to autoimmune and inflammatory diseases (11). Monovalent or divalent targeting of FcRs bearing an ITAM motif induced ITAMi signals that involved activation and recruitment of the Src homology region 2 domain-containing tyrosine phosphatase SHP-1 (Figure 1, Right). It has been demonstrated that other immunoreceptors such as the antigen receptors BCR and TCR can also associate with SHP-1 upon interaction with low avidity ligands (15, 16). Moreover, SHP-1 deficiency in hematopoietic cells favors development of various auto-immunes diseases. For example, the motheaten mice (mev/mev) which express approximately 20% wild type activity of SHP-1, develop severe immune dysregulation and autoantibody production (17).

During ITAMi signaling induced by FcRs, Lyn is essential for the phosphorylation (on tyrosine residue 536) and the activation of SHP-1 (4). It has been reported that Lyn is involved in positive and negative signals induced by antigen receptors (18, 19). Lyn plays an important role in the negative selection of B cells in the bone marrow, since the absence of Lyn was associated with a decreased B cell number in the periphery of mice. In the absence of Lyn, other SFKs, such as Fyn, act as positive regulators of BCR signaling, suggesting a loss of anergy. The opposite roles of Lyn and Fyn were recently demonstrated by *in vivo* approaches. Lyn deficiency aggravates auto/inflammatory diseases such as nephritis and arthritis, while the absence of Fyn protects against these diseases (4). Additionally, we showed that activation of leukocytes in lupus nephritis patients was associated with Fyn-activated signature, suggesting that the balance between Lyn and Fyn is dysregulated during diseases.

Another FcR that play an essential role in the transcytosis by epithelial cells of dimeric IgA, but also pentameric IgM (notably during IgA deficiencies), is called the polymeric immunoglobulin receptor (pIgR). The pIgR is internalized with its ligands by endocytosis and transcytosed from the basolateral membrane into apical side of the epithelial cell (20). The central role of this receptor is to generate secretory IgA (formed of IgA dimers linked to the extracellular domain of the pIgR, also known as secretory component) in exocrine secretions to establish host-microbiota symbiosis and to mediate the protection of mucosal surfaces against pathogens (20, 21). The Fcα/μR, the Fc receptor for IgA and IgM, may play a role in systemic and mucosal immunity. It has been shown that none of the B cells, T cells, monocyte/macrophages, or NK cells in human blood samples expressed this receptor irrespective of age, ethnic origin or gender. Its expression is restricted to B cells from germinal center, follicular dendritic cells and tonsillar cells. Although, the exact function of the Fcα/μR is not fully clarified, it may play an important role in antigen presentation and B cell selection in the germinal center responses (22).

**Figure 2:**
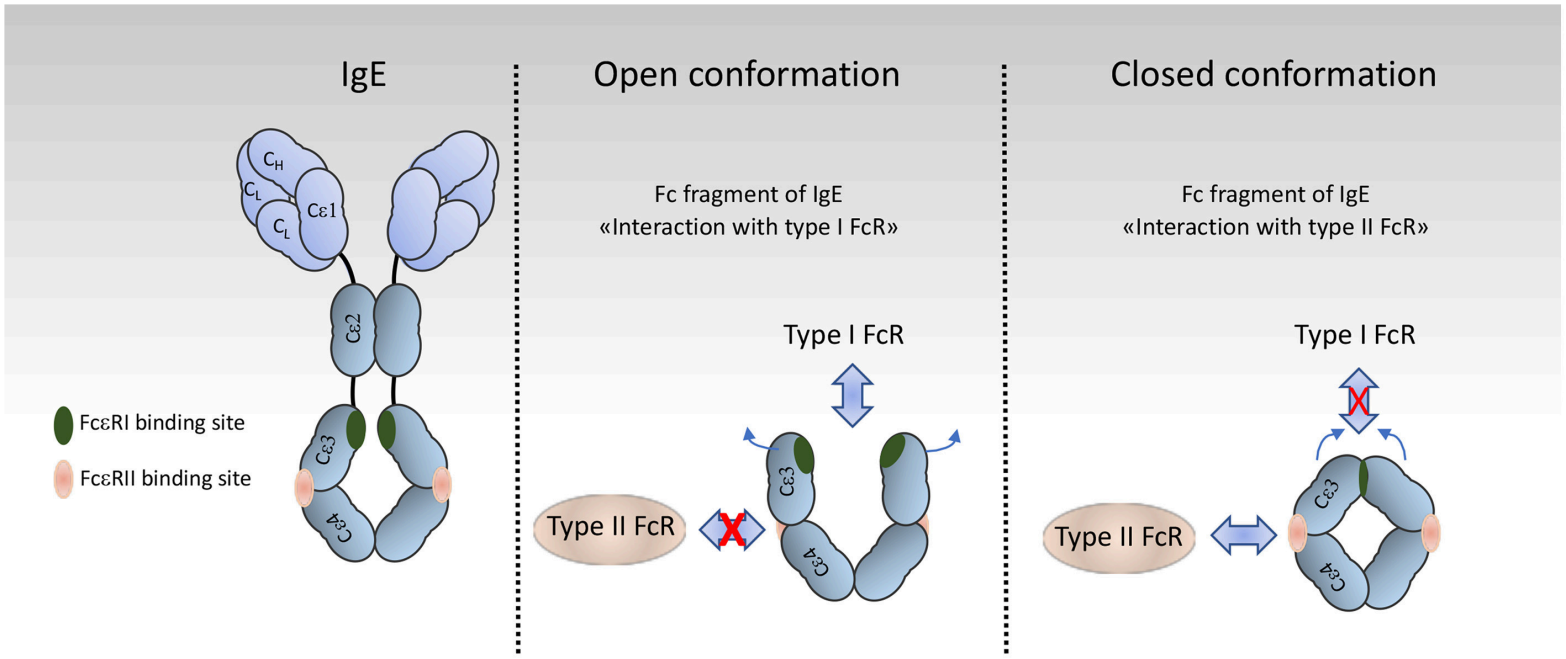
Organization and conformational rearrangements of the IgE Fc. **(left)**, IgE and the binding sites to FcεRI (green) and to CD23 (pink) [adapted from Pennington et al. (24)] **(Middle** and **Right)**. Representation of the open and closed conformations, respectively, of the IgE Fc Cε3–4 domains, and the mutual allosteric inhibition by FcεRIα (green) and CD23 (pink).

**Figure 3:**
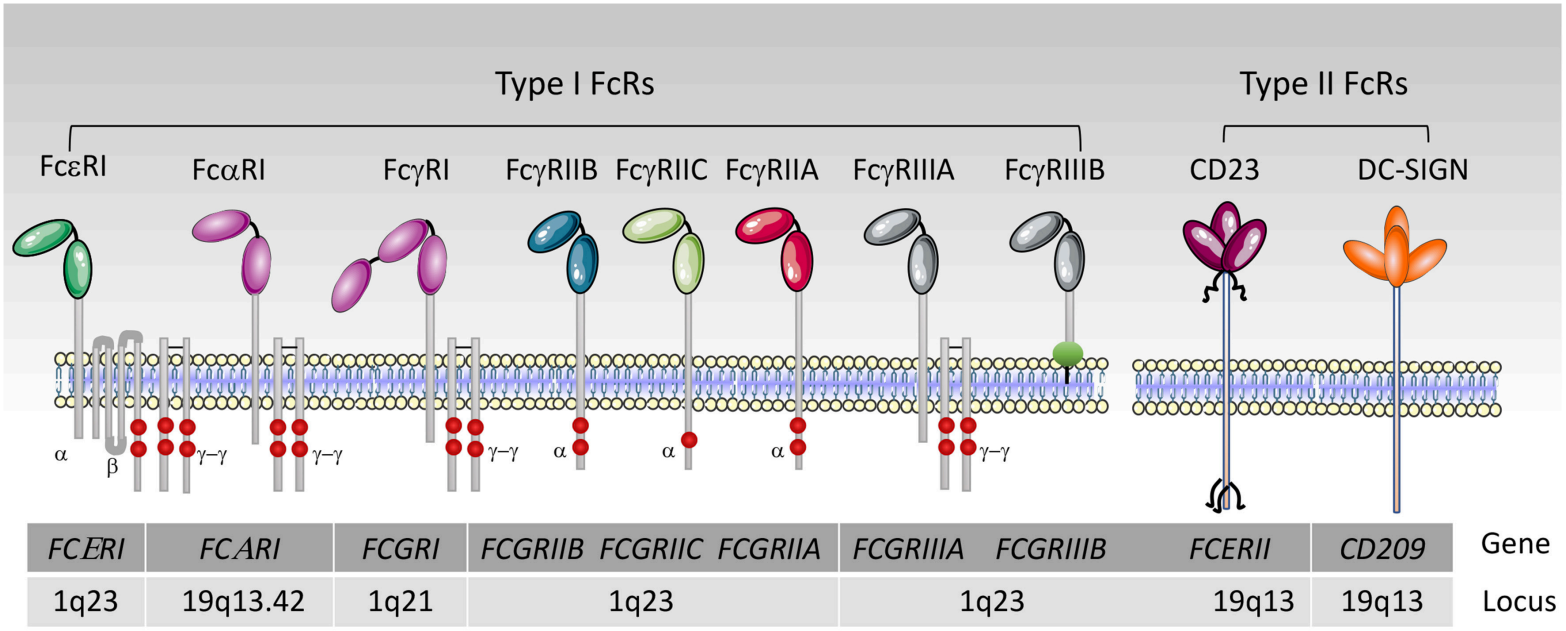
Human type I and II Fc receptors. Schematic representation of human Fc receptors at the cell membrane and their association or not with the FcRγ-chain dimer and the FcεRIβ chain, red circles represent the tyrosine residues. The FcγRIIIB is anchored into the outer leaflet of the plasma membrane by a phosphatidylinositol-glycan (green circle).

FcRs are divided into type I and type II on the basis of the conformational state of the Ig Fc domain that interacts with the receptor (1, 23). Type I Fc receptors interact with “open,” but not “closed” Ig Fc conformation (Figure 2). These receptors include FcγRI, FcγRII, FcγRIII, FcεRI, FcαRI, FcμR, and Fcα/μR (25– 29). In contrast, type II FcRs, bind preferentially Ig Fc domains in “closed” conformation. Among these are C-type lectin receptors such as FcεRII (CD23) and DC-SIGN (Figure 3).

For type II Fc receptors, glycosylation of the Fc domain induces a conformational change that occludes the binding site for type I Fc receptors that lies near the hinge region (open conformation) and reveals a binding site at the CH2-CH3 domain interface (closed conformation). These receptors bind antibodies in a two receptors-to-one antibody stoichiometry that may influence signal initiation (1). DC-SIGN and SIGN-R1, for example, bind secretory IgA and play an intriguing role in dendritic cells inducing IL10 and Treg-mediated tolerance (30). Signaling through these receptors, however, is not yet documented as compared to type I FcRs, with the exception of CD23. Crosslinking of CD23 on B cells activates cAMP (31) and intracellular calcium flux (32) which is associated with the activation of the SFK Fyn and of the PI3K pathway (33). These findings are in agreement with our recent data on type I FcRs (4), and indicates that Fyn also plays an activating role in B cells through type II Fc receptors.

## Fc Receptors and Diseases

### Gene Alleles

Several single-nucleotide polymorphisms (SNPs) have been reported in the genes encoding activating FcγRs (FcγRIIA, FcγRIIIA, and FcγRIIIB). In the gene encoding the inhibitory FcγRIIB, a SNP has been described which is associated with autoimmune diseases such as SLE and rheumatoid arthritis (RA) (34, 35). In addition to SNPs, copy-number variations (CNVs) of FcγR genes are associated with susceptibility to autoimmune disorders (34–40). Most polymorphisms concern the extracellular domains which bind to IgG, affecting the affinity between these receptors and IgG subclasses. However, no polymorphism and CNV have been clearly identified for FcγRI.

The most studied polymorphism is the one in the second Ig-like extracellular domain of the FcγRIIA that results in a point mutation of amino acid at position 131, coding for either arginine (R131) or histidine (H131). FcγRIIA-R131 binds less efficiently IgG2 than FcγRIIA-H131 (34). This *Fcgr2a* polymorphism has been described as a heritable risk factor for autoimmune and infectious diseases (41, 42). Moreover, genome-wide association studies (GWAS) revealed that FcγRIIA-H131 variant is associated with higher susceptibilities to develop Kawasaki disease and ulcerative colitis (43, 44). This variant is also associated with Guillain–Barré syndrome (45), supporting that immune complexes that include IgG2 auto-antibodies are involved in inflammatory responses. In contrast, the genotype homozygous for the FcγRIIA-R131 variant-encoding gene is associated with SLE, immune thrombocytopenic purpura (ITP) and IgA nephropathy (IgAN), revealing a complex and contrasted picture for the role of IgG2-containing immune complexes in autoimmune diseases (46–48). Regarding infectious diseases, neutrophils homozygous for the gene encoding the H131 variant show a higher capacity for IgG2-mediated phagocytosis than those homozygous for the gene encoding the R131 variant (41). In agreement, patients with the R encoding allele were found to have more severe cases of Severe Acute Respiratory Syndrome infection and were more susceptible to encapsulated microorganism infection, which was attributed to poor IgG2 binding to the R131 variant of FcγRIIA (41, 42). As mice do not express FcγRIIA homologs, transgenic mice expressing the *Fcgr2a* human gene encoding the R131 variant develop spontaneously autoimmune diseases such as pneumonitis, glomerulonephritis and RA (49, 50). Moreover, the fact that FcγRIIA-R131 expressed on the FcRγ-/- background in mice similarly develop thrombocytopenia (51) and arthritis (11, 52) indicates that pro-autoimmune signals through FcγRIIA-R131 ITAM were sufficient to induce diseases. Biochemical analyses showed that two tyrosines of FcγRIIA ITAM motif were needed to induce inflammatory signals (53). Taken together, these animal models underline the critical involvement of *Fcgr2a* polymorphism in a number of diseases.

A polymorphism has been found in the inhibitory FcγRIIB-encoding gene that leads to a single I-to-T amino acid substitution in the transmembrane domain (residue 232) (43, 54). Human monocytes expressing the FcγRIIB-T232 failed to inhibit heterologous receptors-mediated cell activation (55). The FcγRIIB-T232 polymorphism is associated with susceptibility to develop auto-immune diseases such as SLE (42, 54, 56). However, there are some discordances concerning the SNPs in the promoter region of *Fcgr2b*, such as −386G and −120T (haplotype 2B.2), and −386C and −120A (haplotype 2B.4) variants. It has been shown that the 2B.4 SNP promoter haplotype upregulates the expression of FcγRIIB on neutrophils and monocytes that negatively correlates with lupus nephritis (46). This is in agreement with previous reports in mouse SLE-like models and suggests that FcγRIIB expression is protective in SLE (57). However, in striking contrast and in an apparent paradox, the same 2B.4 promoter haplotype was found by the same authors to correlate with SLE (46). This positive (SLE) vs. negative (lupus nephritis) paradoxical association of the 2B.4 promoter haplotype suggests multifaceted impacts of FcγRIIB in SLE that may depend on the affected cell types (e.g., monocytes *vs*. neutrophils). Alternatively, particular cell types expressing FcγRIIB could have aggravating or protective actions in SLE depending on which affected tissue these cells are recruited to, or on how these cells impact the systemic *vs*. local aspect of the disease. Thus, further investigation is necessary to elucidate the association of the promoter haplotype in disease development.

The *Fcgr3a* polymorphism is characterized by a point mutation in the codon for residue 158, encoding valine (V158) or phenylalanine (F158) in the Ig-like domain near the membrane (34–36). The FcγRIIIA-V158 variant has a higher affinity for all human IgGs than the FcγRIIIA-F158 variant (40). The FcγRIIIA-F158 is associated with susceptibility to SLE, Crohn's disease and Behçet's disease (35, 36). Although studies have also explored the association between RA and the V or F 158 variant, their results have been contradictory and this question remains unsettled (58, 59).

Several *Fcar1* polymorphisms have been found, including two in the functional promoter region of the FcαRI encoding gene (−114T/C and +56T/C relative to the major transcription start site) (60). The incidence of the −114C/C polymorphism in patients with IgAN was significantly increased compared with other chronic kidney diseases (CKD) and healthy donors (HD) (15.6 vs. 4.0% in other CKD and vs. 2.4% in HD). This *Fcar1* polymorphism in the promoter region appears to be associated with susceptibility to IgAN, suggesting the importance of FcαRI expression in this disease. A third *Fcar1* polymorphism has been described in the coding region for FcαRI, which changes codon 248 from AGC to GGC leading to G248 instead of S248 in the cytoplasmic domain of the receptor (61). Interestingly, these two different alleles demonstrate significantly different FcαRI-mediated intracellular activating signaling. The proinflammatory FcαRI-G248 variant has been associated with SLE in two ethnic groups (61). However, this *Fcar1* polymorphism was not associated with other auto-immune diseases such as systemic sclerosis, RA or IgAN (62, 63). A fourth *Fcar1* polymorphism (A/G at nt 324) was also associated with aggressive periodontitis (64). Patients displaying the nt 324 A/A allele presented polymorphonuclear neutrophil dysfunctions with a decreased phagocytosis of periodontopathic bacteria (Porphyromonas gingivalis) as compared to patients expressing the nt 324 G/G (64).

Regarding the pIgR, it has been reported as a susceptibility gene for nasopharyngeal cancer (NPC) associated with Epstein-Barr virus (EBV) (65). This lead to a hypothesis that pIgR could be the nasopharyngeal epithelial receptor for EBV via IgA-EBV complex. Transcytosis failure due to missense C → T mutation on the *PIGR*1739 nucleotide (resulting in an A-to-V mutation near the endoproteolytic cleavage site of pIgR) could decrease the ability of pIgR to release IgA-EBV complexes, thus increasing susceptibility to develop NPC (65).

The high-affinity FcεRI is expressed by mast cells and basophils and plays an important role in allergic diseases. Several studies have identified two FcεRI polymorphisms associated with allergies. The −66T > C and/or the −315C > T are associated with atopic dermatitis, chronic urticaria, asthma, and high serum IgE levels (66–69). These polymorphisms were also associated with allergic inflammatory diseases such as atopy and nasal allergy (70, 71).

Table 1 summarizes most of described FcR alleles and their expression and functions in physiology and pathology (42–44, 46, 48, 60, 65, 66, 69, 72–83).

**Table 1:** Human FcRs: their expression, function and allotypes.

**Name**	**Subclass binding**	**Expression**	**Functions**	**Alleles**	**Link to diseases**
FcγRI (CD64)	IgG 1/3/4	Monocytes/Macrophages Neutrophils/DCs/Mast cells	Activation	–	–
FcγRIIA (CD32a)	H_131_:lgG 1/2/3/4 R_131_:lgG l/(2) /3/4	Monocytes/Macrophages Neutrophils/DCs/Basophils/Mast eells/Eosinophils	Activation/inhibition	H131/R131	Kawasaki diseases (43), Ulcerative colitis (44). Childhood-onset ITP (71)/Lupus (46), IgAN (48), arthritis
FcγRIIB (CD32b)	lgG l/(2)/3/4	B cells/DCs/Mast cells/Basophils	Inhibition	Promoter−3S6C or −120/T232	Lupus (46)/lupus (42), Atopy (72)
FcγRIIC (CD32c)	IgG 1/(2)/3/4	NK cells/ Monocytes/Macrophages /Neutrophils	Activation	Q13/stopl3	Kawasaki disease (73)
FcγRIIIA (CD16)	V_158_: lgG l(2)/3/4 F_l58_: lgG l/(2)/3/4	NK cells/ Monocytes/Macrophages	Activation/Inhibition	V15S/F158	IgAN (48), arthritis seventy (74), childhood chronic ITP (75)/Lupus (76), arthritis (58), Crohn's disease (77)
FcγRIIIB (CD16b)	IgG 1/3/4?	Neutrophils/ Eosinophils/ Basophils	Activation		Wegener's granulomatosis (78)
FcαRI (CD89)	IgAl, lgA2, CRP	Monocytes/Macrophages Neutrophils/DCs/Kupffer cells (79)	Activation/Inhibition	114T/C	IgA nephropathy (60), AIDS, ankylosing spondylitis, alcoholic liver cirrhosis, Henoth-Schonlem purpura (HSP) (26)
FcμR	IgM	B and T lymphocytes	Inhibition/?		Chronic Lymphocytic Leukemia (CLL) (80, 81)
Fcα**/**μR	IgM and IgA	Germinal center B cell, Follicular dendritic cells	?		
FcεRI	IgE	Mast cells/Basophils	Activation	66T/31SC	Atopic dermatitis._J_ asthma and chronic urticaria (66–71)
FcεRII (CD23)	IgE	B cells and macrophages	Activation	–	AIDS and B-CLL (82)
FcRn	IgG 1/2/3/4	Monocytes/Macrophages Neutrophils/DCs/endothelium/Syncytiotrophoblasts	Recycling Transport uptake	VNTR1–5	
PlgR	plgA	Mucosal epithelium	Transcytosis	1739C to T	Nasopharyngeal cancer and infection (65)

*From left to right columns: names, function, alleles that include amino acid variations in immunoglobulin domains and the transmembrane domain, cellular expression of FcRs, and diseases linked to alleles and CNVs (reference numbers are shown), and binding abilities of IgG subclasses to each FcR allele. IgAN, IgA nephropathy; CLL, chronic lymphocytic Leukemia; FcRn, neonatal Fc receptor; AIDS, acquired immune deficiency syndrome; PIgR, Polymeric immunoglobulin receptor; DCs, dendritic cells*.

### Alterations in FcR Expression

Several studies highlighted altered expression, structure and function of FcγRs in patients. Whereas, CD4^+^ T cells from healthy donors fail to express significant levels of FcγR, FcγRIIA is expressed in a subpopulation of CD4^+^ T cells in blood samples from HIV-1-positive patients and is highly enriched in inducible replication-competent proviruses suggestive of an FcγRIIA^+^ HIV reservoir (84). Yet, in other studies, FcγRIIA expression did not selectively enrich for HIV- or SIV-infected CD4^+^ T cells in peripheral blood or lymphoid tissue since resting FcγRIIA^+^ CD4^+^ T cells have <3% of the total HIV DNA amongst CD4^+^ T cells (85, 86). Taken together, whereas FcγRIIA expression in CD4^+^ T cells becomes a marker for HIV infection, the involvement of FcγRIIA^+^ CD4^+^ T cells in AIDS remains to be elucidated.

The inhibitory FcγRIIB, in contrast, is down-regulated in autoimmune diseases notably on both memory and plasma B lymphocytes of active SLE patients compared to those from healthy individuals (87). However, this down-regulation was not seen on myeloid-lineage cells. This was also observed in Hashimoto's thyroiditis (88).

High level of FcγRIIIA expression on monocytes together with that of CD14 is associated with proinflammatory cytokine profiles and higher potency in antigen presentation allowing to define monocyte subsets with distinct phenotypes and functions (89).

For FcαRI, its expression is dysregulated in patients with AIDS, ankylosing spondylitis, alcoholic liver cirrhosis, Henoch-Schonlein purpura (HSP) and IgAN (90–93). Some of these studies have shown biochemical abnormalities revealing FcαRI altered protein mobility in SDS-PAGE suggesting altered glycosylation of this receptor (90). Interestingly, mutational studies of FcαRI indicate that the N58 residue of the receptor controls IgA-binding enhancement (94). In parallel, abnormally glycosylated IgA1 molecules (hypogalactosylation and hyposialylation on the hinge region) observed in patients with IgAN and HSP is associated with the shedding of a soluble form of FcαRI (sFcαRI), which participates in the formation of circulating IgA1 complexes (95). These IgA1-sFcαRI complexes were decreased in serum of IgAN patients with severe and progressive disease as compared to non-progressive IgAN patients (96) suggesting a kidney deposition, and hence a possible nephrotoxic action, of such complexes which is further supported by studies in IgAN patients with recurrence of the disease after kidney transplantation (97). In this study, IgA-sFcαRI complexes were decreased in the serum of patients with recurrent IgAN and sFcαRI was detected in the kidney mesangium only in patients with the recurrent disease. Direct evidence for a nephrotoxic role of IgA1-sFcαRI complexes were obtained in humanized animal models. These experimental studies were based on the fact that mouse do not have homologs of IgA1 and FcαRI. Humanized mice expressing human IgA1 and human FcαRI spontaneously develop mesangial deposits of IgA1-sFcαRI complexes (98). In the glomeruli, these complexes are captured by the transferrin receptor 1 (TfR1), which is upregulated on mesangial cells, through interaction with polymeric (p) IgA1 and FcαRI (98, 99). Although the mechanism of TfR1 upregulation remains poorly understood, the crosslinking enzyme transglutaminase 2 has been found to be overexpressed and associated with the receptor controlling mesangial IgA1 complex deposition and renal injury (98). Polymeric IgA1 induce TfR1 expression *in vitro* on mesangial cells. Polymeric IgA1-TfR1 interaction triggers activating signals through mTOR, PI3K and ERK pathways, and phosphorylated ERK is associated with disease progression (100). Interestingly enough, in physiology TfR1-IgA1 interaction plays a role in erythropoiesis (101). Progression of IgAN to end-stage renal disease may also involve FcαRI activation on tissue macrophages surrounding hypogalactosylated IgA1-mediated mesangial lesions. Indeed, FcαRI_R209L_Tg mice, with an R-to-L substitution at position 209 in the transmembrane region of FcαRI, did not develop macrophage infiltration and proteinuria (102). This mutant receptor cannot associate with the ITAM-bearing FcRγ signaling subunit (103). In agreement, only macrophages expressing wild-type FcαRI, but not those expressing FcαRI_R209L_, were able to migrate to the kidney after adoptive transfer demonstrating that their chemotaxis depends on the FcRγ subunit. Of note, FcαRI can be found associated and non-associated with FcRγ on the same cells (95). Since mouse IgA and human FcαRI interaction may be sufficient to induce receptor shedding leading to IgA deposits in the kidney, we hypothesized that both receptor types could cooperate to induce disease, the FcRγ-less FcαRI allowing IgA deposits and the FcRγ-associated FcαRI promoting inflammatory cell infiltration and disease progression (102).

For FcμR (IgM receptor), deficiency in the receptor in mice revealed that this receptor plays a crucial role in B cell responses (27, 104). Mice deficient for *Fcmr* are characterized by the increase in pre-immune serum IgM, dysregulation of humoral immune responses, disturbances in B cell subpopulations, B cell proliferation alteration after BCR ligation, and autoantibody production (104, 105). Accordingly, in chronic lymphocytic leukemia (CLL), the membrane expression and the soluble form of FcμR in serum were increased. The potential mechanism proposed for the up-regulation of FcμR is that the antigen-independent self-ligation of BCR on CLL cells induces activation of Syk thus increasing the cell surface expression of FcμR. Furthermore, the IgM antibodies produced by CLL cells that had differentiated into plasma cells, recognized soluble or lymphocyte membrane self-antigens. IgM/self-antigen immune complexes would then crosslink FcμR and BCR favoring cell survival. An alternative splice variant of the soluble FcμR is increased in CLL patients, but its biological function is unclear (105, 106).

For type II FcRs, an increased expression of FcεRII on monocytes in AIDS patients has been associated with the aberrant activated phenotype of these cells during the immunopathogenesis of AIDS. Interestingly, despite the known ability of IL-10 to downregulate monocyte FcεRII expression, in AIDS the IL-10-enriched environment is not associated with the suppression of FcεRII expression on monocytes (82) indicative of an impairment of this negative regulation in patients. In B-CLL also, patients strongly express FcεRII, which is associated with B cell activation and proliferation. Moreover, altered phosphorylation of FcεRII intracellular tail were reported in B-CLL B lymphocytes (107) further supporting an active role of FcεRII in this disease.

## Targeting of Fc Receptors as Therapeutic Approaches

### Blocking/neutralizing Activating Receptor

#### Antibodies

Both murine models and studies in patients suggest a major role of the activating FcRs in initiating and propelling immune complex-mediated inflammatory reactions. For example, human FcγRIIA transgenic mice are hypersensitive to pathogenic antibodies and develop destructive arthritic syndromes. *Ex vivo* experimentation with circulating monocytes from RA patients suggest that FcγRIIA is responsible for the production of reactive oxygen species (11, 108). Anti-receptor monoclonal antibodies, intact antibodies and antibody fragments as well as a variety of small molecules have been designed to interact with the Ig-binding domains in activating FcRs. Some of these approaches have shown encouraging results when tested *in vitro* or *in vivo* for blocking immune complex-mediated cell effects and inflammation. Recently, we have demonstrated that divalent targeting of FcγRIIA by anti-hFcγRII F(ab)′2 fragments ameliorates RA-associated inflammation. This therapeutic effect was mediated by the induction of inhibitory ITAM (ITAMi) signaling through the activation of SHP-1. Moreover, treatment of inflammatory synovial cells from RA patients by F(ab′)2 fragment of hFcγRIIA-specific antibody inhibited production of reactive oxygen species associated with the induction of FcγRIIA-mediated ITAMi signaling. These data suggest that targeting of hFcγRIIA by specific antibody such as clone IV.3 mAb could ameliorate RA-associated inflammation (11). Anti-FcαRI Fab and F(ab)′2 fragments also have demonstrated efficiency on RA (109). Interestingly, in autoimmune blistering skin diseases that involve interaction between IgA autoantibodies and the neutrophil FcαRI, targeting FcαRI by blocking peptides or antibodies prevents neutrophil migration and tissue damage *ex-vivo* (110, 111).

In allergy, treatment by anti-IgE antibodies has been considered a therapeutic option for a long time. The recombinant anti-IgE humanized monoclonal antibody-E25, named “omalizumab,” is now used in several clinical trials and shows efficacy against IgE-mediated allergic reactions (112, 113) through inhibition of IgE binding to FcεRI on the surface of mast cells and basophils (113).

The above-described upregulation of FcμR expression in CLL cells is of significant clinical interest. It can be easily evaluated by flow cytometry on cells and, additionally, the levels of soluble FcμR may correlate with disease progression. Thus, it may be used as a new biomarker for CLL (106). FcμR is a good target also because it is involved in the pathogenesis of CLL and in the progression of the disease through support of leukemic cell survival (80). Hence, disrupting CLL survival signals might be achieved through FcμR therapeutic targeting. However, a large cohort of CLL patients will be required to validate these two applications (106).

#### IVIG

Intravenous immunoglobulins (IVIG) are harvested from the pooled plasma of 3,000 to 100,000 healthy donors. They consist of over 95% IgGs with a subclass distribution corresponding to that found in normal human serum (114). IVIG is used in treatment of several immunodeficiency diseases including idiopathic thrombocytopenic purpura (ITP), Kawasaki disease, and neurologic diseases such as Guillain–Barre syndrome, chronic inflammatory demyelinating polyneuropathy, myasthenia gravis, sclerosis, and autoimmune encephalitis (115). In ITP patients, administration of IVIG can efficiently attenuate platelet clearance from the circulation. The first proposed mechanism was the competitive blockage of the activating FcγRs on myeloid cells by IVIG, which in turn decreases autoantibody-mediated platelet phagocytosis and ADCC against platelets (116). Furthermore, in pediatric ITP patients, intravenous administration of the Fcγ fragments prepared from IVIG resulted in a rapid recovery in platelet counts (117) further indicating the role of FcγRs in IVIG action. Another IVIG anti-inflammatory mechanism involves saturation of FcRn, the IgG recycling receptor (118). FcRn plays an important role in the maintenance of IgG half-life. Therefore, inhibition of autoantibody activity can be induced by the alteration of their interaction with FcRn, impairing their half-life and accelerating their clearing from the circulation. IVIG by competing with autoantibodies for FcRn binding could therefore facilitate their clearing.

A role for the inhibitory FcγRIIB has been proposed to be exclusive in IVIG action to explain their Fc dependent effect (118). This statement was based notably on studies showing a decreased anti-inflammatory effect of IVIG in FcγRIIB-deficient animals. In other studies, a role for FcγRIII in IVIG-mediated inhibition has been reported (119) although the mechanism of action was not clearly established. Recently, we reported that IVIG can control inflammatory responses by ITAMi signaling through FcγRIIA and FcγRIII (10, 11). These data are based on the *in vitro* targeting of FcγRIIA and FcγRIII by IVIG at the physiological concentration of IgG showing an inhibitory effect on endocytosis. This was confirmed by targeting FcγRIIA or FcγRIII with F(ab′)2 fragments of specific antibodies. These results were further supported *in vivo* in mice by targeting these receptors with IVIG or with specific antibodies and this inhibitory effect was abolished in receptor-deficient mice (10, 11). Therefore, IVIG could use a combination of non-exclusive mechanisms to promote protection against auto-immune diseases. Although IVIG is well tolerated, some patients develop immediate or delayed adverse effects depending on the time occurrence. The Flu-like symptoms such as fever, fatigue and nausea are the most frequent adverse effects. For the delayed adverse effects, the most frequent are thrombotic events, neurological disorders and renal failure. These delayed adverse effects are rare but dangerous (120). The majority of adverse effects are associated with high doses of immunoglobulins; thus, determining individual dosages to guarantee the efficacy of therapy and minimize adverse effects is an urgent goal.

Treatment with highly purified serum monomeric IgA (mIgA) decreases cell activation through FcαRI-FcRγ-mediated ITAMi signaling (109). Human mIgA or anti-FcαRI Fab fragments were used to prevent or treat collagen antibody-induced arthritis in FcαRI-transgenic mice. mIgA treatment decreased significantly leukocyte infiltration to the inflamed joints of mice, which was associated with SHP-1 phosphorylation at Y536 residue in joint tissue cells. Moreover, mIgA reversed the activating ITAM to ITAMi signature and the state of inflammation in the synovial fluid isolated from RA patients (109). Of note, protection was also achieved with human serum IgA (4). These findings open new avenues to develop the concept of IVIgA as a new treatment option for inflammatory and auto-immune diseases.

### Engagement of the Inhibitory FcγRIIB (Agonist)

The only FcR containing an inhibitory ITIM motif, FcγRIIB, serves as a critical negative regulator in immune complex driven reactions. In mice lacking FcγRIIB auto-immune symptoms are exacerbated, and a partial restoration of FcγRIIB expression in B cells rescued mice from developing an SLE-like phenotype (57, 121). Several FcγRIIB specific mAbs have now been developed (122, 123), one of which, mAb2B6, has been chimerized and humanized to direct myeloid-cytotoxicity against B cells (123). These antibodies have the potential to serve as novel immune suppressors in auto-immunity either by blocking B cell activation or by targeting their destruction. In addition, they may have an advantage over CD20 antibodies for their ability to target plasma cells (124).

### Targeting FcRn

Blocking FcRn-IgG interaction to decrease circulating IgG levels is one strategy to treat auto-immune disease (118). In the absence of interaction with FcRn, IgG would be degraded in lysosomes more quickly instead of being recycled back into circulation. One straightforward method would be to use recombinant soluble human FcRn to compete with membrane FcRn for IgG. Another approach to block IgG-FcRn binding would be through engineered “bait” IgG which occupy FcRn thus preventing binding of endogenous IgG. Such “bait” antibodies have been generated with a much higher affinity for FcRn at both acidic and neutral pH, thereby providing effective occupancy of FcRn, competing with, and resulting in, degradation of endogenous IgG. These antibodies are also called “Abdegs”: antibodies that enhance IgG degradation (125).

An FcRn-specific blocking mAb would also provide interference with FcRn-IgG interaction. One such mAb, 1G3, was examined in rat passive and active models of myasthenia gravis, a prototypical antibody-mediated auto-immune disease (126). Treatment by 1G3 mAb resulted in amelioration of disease symptoms in a dose-dependent manner together with greatly reduced levels of pathogenic antibody in the serum.

### Other Future Strategies to Target FcR-Effectors to Treat Auto-Immune/Inflammatory Diseases

Targeting of FcRs by monomeric immunoglobulins or by F(ab′)2 fragments of specific antibodies, induces ITAMi signaling which involved the recruitment of Lyn, but not Fyn. The Src kinase Lyn, leads to partial phosphorylation of the ITAM motif on tyrosine residues (11), and to the conformational change of SHP-1 that allows its recruitment through its SH2 domains to ITAM phosphotyrosine residues (127). This recruitment induces a Lyn-dependent phosphorylation of SHP-1 on Y536 and to SHP-1 phosphatase activity that inhibits the recruitment of various proteins induced by heterologous receptors (52). In contrast, multivalent crosslinking of immunoreceptors by immune complexes induces the recruitment of both Src kinases Lyn and Fyn to the receptor leading to full phosphorylation of the tyrosine residues present in the ITAM motif. This leads to the activation and the recruitment of the kinase Syk. In parallel, Fyn initiates a signaling pathway involving a PI3K-PKCα axis leading the inactivation of SHP-1 through the phosphorylation of its S591 residue barring its recruitment to the plasma membrane (128). Since S591 phosphorylation on SHP-1 keeps the phosphatase in a closed conformation (127), our recent study showed that the phosphorylation of SHP-1 on S591 residue by Fyn axis renders the Y536 residue inaccessible to Lyn. In agreement, the absence of Fyn favors the phosphorylation of SHP-1 on Y536 by Lyn, despite the crosslinking of FcγRIIA (4). These results suggest that the selective absence or inhibition of Fyn may abolish inflammation during auto-immune and proinflammatory diseases. Taken together, inhibition of Fyn or of the molecules which are upstream or downstream this SFK reverses inflammation during auto-immune and inflammatory diseases and thus, could be a new therapeutic strategy to decrease the activating ITAM signaling in these diseases. Along these lines, inhibition of PI3K (a major player of the Fyn-PI3K-PKCα axis (4)) prevents RA and lupus nephritis progression in mouse models (129). However, it should be mentioned that since Fyn is essential for activating ITAM signals (i.e., phagocytosis), the inhibition of this SFK may favor infections. Moreover, Fyn plays also other roles independently of FcRs. It has been shown that the absence of Fyn impaired multipolar-bipolar transition of newly generated neurons and neurite formation during the early phase of migration. Additionally, inhibition of Fyn decreased the branching number of the migrating cortical neurons (130). Another important hurdle is that Lyn and Fyn present a high homology and there are currently no selective inhibitory drugs. Therefore, Fyn does not appear as the best target to treat auto-immune and pro-inflammatory diseases. Identification of new targets which are downstream of Fyn and which are expressed specifically by immune cells involved in auto-immune diseases will permit development of new therapeutic strategies for auto-immune diseases that involve FcR.

## Conclusion

Fc receptors may be responsible for diseases when dysregulated in spite of their physiologic protective function. Unraveling all aspects (expression, function, regulation) of FcR biology should help to define approaches to correct the first and to wield the second to restore homeostasis thus representing new hopes for innovative anti-inflammatory strategies. Progress in these two aspects is currently well underway, already proposing new potent therapeutic tools. The future in this field is a promise of scientific excitement.

## Author Contributions

SB and RM wrote this review. MB has critically read the manuscript.

### Conflict of Interest Statement

The authors declare that the research was conducted in the absence of any commercial or financial relationships that could be construed as a potential conflict of interest.
